# Rhizobia with different symbiotic efficiencies nodulate *Acaciella angustissima* in Mexico, including *Sinorhizobium chiapanecum* sp. nov. which has common symbiotic genes with *Sinorhizobium mexicanum*

**DOI:** 10.1111/j.1574-6941.2008.00590.x

**Published:** 2008-10-01

**Authors:** Reiner Rincón-Rosales, Lourdes Lloret, Edith Ponce, Esperanza Martínez-Romero

**Affiliations:** 1 Departamento de Biotecnología Vegetal, Instituto Tecnológico de Tuxtla Gutiérrez, Tuxtla GutiérrezChiapas, Mexico; 2 Centro de Ciencias Genómicas, Universidad Nacional Autónoma de MéxicoCuernavaca, Morelos, Mexico

**Keywords:** *Acaciella angustissima*, N_2_ fixation, nodulation, rhizobial diversity, legume symbiosis

## Abstract

Bacteria from nodules of the legume *Acaciella angustissima* native to the south of Mexico were characterized genetically and their nodulation and competitiveness were evaluated. Phylogenetic studies derived from *rpoB* gene sequences indicated that *A. angustissima* is nodulated by *Sinorhizobium mexicanum, Rhizobium tropici, Mesorhizobium plurifarium* and *Agrobacterium tumefaciens* and by bacteria related to *Sinorhizobium americanum, Sinorhizobium terangae, Rhizobium etli* and *Rhizobium gallicum*. A new lineage related to *S. terangae* is recognized based on the sequences of *gyrA, nolR, recA, rpoB* and *rrs* genes, DNA–DNA hybridization and phenotypic characteristics. The name for this new species is *Sinorhizobium chiapanecum* and its type strain is ITTG S70^T^. The symbiotic genes *nodA* and *nifH* were similar to those from *S. mexicanum* strains, which are *Acaciella* symbionts as well, with *nodA* gene sequences grouped within a cluster of *nod* genes from strains that nodulate plants from the Mimosoideae subfamily of the *Leguminosae. Sinorhizobium* isolates were the most frequently obtained from *A. angustissima* nodules and were among the best strains to promote plant growth in *A. angustissima* and to compete in interstrain nodule competition assays. Lateral transfer of symbiotic genes is not evident among the genera that nodulate *A. angustissima* (*Rhizobium, Sinorhizobium* and *Mesorhizobium*) but may occur among the sympatric and closely related sinorhizobia that nodulate *Acaciella*.

## Introduction

Bacteria in the roots or the stems of legumes fix nitrogen and provide the plant with this nutrient. Symbiotic bacteria have been studied from only a small proportion of the extant legume species, and diverse genera such as *Rhizobium, Sinorhizobium, Mesorhizobium, Bradyrhizobium, Devosia, Methylobacterium, Burkholderia* and *Cupriavidus* have been reported to contain nodulating species (Young & Haukka, 1996; Sprent, 2001; Sy *et al.*, 2001; Rivas *et al.*, 2002; Chen *et al.*, 2003; Vandamme & Coenye, 2004; Elliott *et al.*, 2007a,b;). Mexico is a very diverse country and occupies the fourth place in plant diversity terms (Rzedowsky, 1978) with many endemic legumes. The genus *Acaciella* is found mainly in Mexico and has well-supported botanical differences to be recognized as a new species different from the *Acacia* genus where it was formerly classified (Rico-Arce & Bachean, 2006).

Nitrogen-fixing trees and shrubs are valuable to maintain forest fertility and N_2_ fixation allows their growth in infertile soils while enriching soil nitrogen. In Chiapas, *Acaciella angustissima* shrubs that can grow in poor soils are being used in agroforestry systems, due to their rapid growth rate, high capacity for nitrogen fixation and the quality of the tannins that accumulate in their bark (Rincón-Rosales & Gutiérrez-Miceli, 2008). Interestingly, these shrubs are the preferred hosts of *Llaveia mexicanorum* (Williams & MacVean, 1995), a native homeoptera scale insect, which is used by indigenous people of Chiapas and Mesoamerica to produce a fat for traditional lacquer wood handcrafts (Grillasca, 2007). We established nurseries to propagate *A. angustissima* plants and became aware that inoculants were required to attain good plant development. This prompted us to analyze and select strains for inoculation.

One of the sinorhizobial groups we encountered corresponded to a new species and we proposed the name *Sinorhizobium mexicanum* for this lineage (Lloret *et al.*, 2007). However, modifications in the *Sinorhizobium* genus taxonomy have occurred (Young, 2003). The bacteria belonging to *Sinorhizobium* have been transferred to the genus *Ensifer* (Young, 2003) because according to judicial rules, *Ensifer* has priority over *Sinorhizobium*. This new *Sinorhizobium* species had to be named as *Ensifer mexicanus* (Lloret *et al.*, 2007) instead of *S. mexicanum*. In this work, we chose to use the former name *Sinorhizobium* as used in many recently published papers.

*Sinorhizobium mexicanum* was not the only symbiont found in *A. angustissima* nodules. The objective of this study was to characterize the other symbionts of *A. angustissima* in Mexico (including a novel sinorhizobial species), their interstrain nodulation competitiveness and their plant growth promotion in *A. angustissima*.

## Materials and methods

### Sample sites

Isolates used in this study were obtained from root nodules of *A. angustissima* collected from the Sumidero Canyon National Park in Chiapas, Mexico, and from nodulated trap plants grown in pots containing soils collected from an ecological reserve area in Sierra de Huautla in Morelos, Mexico (Supporting Information, Fig. S1). The Chiapas and Morelos collecting sites were *c*. 1000 km apart and both are characterized by deciduous forest vegetation (Lloret *et al.*, 2007).

### Bacterial strains

The *A. angustissima* strains analyzed in this study are listed in Table 1. Bacteria were obtained as described by Vincent (1970) using peptone yeast agar (PY) as growth medium (Toledo *et al.*, 2003). Plates were incubated aerobically at 28 °C for 3 days and the isolates were purified by streaking single colonies on fresh PY plates. Single colony formation and morphology were observed in yeast extract mannitol (YEM) and PY media at 28 °C as reported by Toledo *et al.* (2003). The acid/alkaline reaction was verified by spreading the inoculum on YEM plates (pH 7.0) containing 25 μg mL^−1^ bromothymol blue (Vincent, 1970).

**Table 1:** Bacteria isolated from nodules of *Acaciella angustissima*

Species and strains*	Geographical origin
*Agrobacterium tumefaciens*	
ITTG S2	Chiapas, Mexico
ITTG S6	Chiapas, Mexico
ITTG S9	Chiapas, Mexico
ITTG S10	Chiapas, Mexico
CFN ESH11	Morelos, Mexico
CFN ESH16	Morelos, Mexico
*Mesorhizobium plurifarium*	
CFN ESH5	Morelos, Mexico
CFN ESH18	Morelos, Mexico
CFN ESH19	Morelos, Mexico
CFN ESH22	Morelos, Mexico
CFN ESH26	Morelos, Mexico
*Rhizobium* sp. (*R. gallicum* related)	
ITTG S11	Chiapas, Mexico
*Rhizobium* sp. (*R. leguminosarum/R. etli* related)	
CFN ESH6	Morelos, Mexico
CFN ESH7	Morelos, Mexico
CFN ESH34	Morelos, Mexico
*Rhizobium tropici*	
CFN ESH9	Morelos, Mexico
CFN ESH10	Morelos, Mexico
CFN ESH23	Morelos, Mexico
CFN ESH25	Morelos, Mexico
CFN ESH27	Morelos, Mexico
CFN ESH29	Morelos, Mexico
ITTG S7	Chiapas, Mexico
*Sinorhizobium* sp. (*S. americanum* related)	
ITTG S8	Chiapas, Mexico
*Sinorhizobium chiapanecum* sp. nov.	
ITTG R11	Chiapas, Mexico
ITTG S1	Chiapas, Mexico
ITTG S68	Chiapas, Mexico
ITTG S70^T^	Chiapas, Mexico
ITTG S71	Chiapas, Mexico
*Sinorhizobium mexicanum*	
CFN ESH1	Morelos, Mexico
CFN ESH2	Morelos, Mexico
CFN ESH3	Morelos, Mexico
CFN ESH4	Morelos, Mexico
ITTG R4	Chiapas, Mexico
ITTG R7^T^	Chiapas, Mexico
ITTG S3	Chiapas, Mexico
ITTG S4	Chiapas, Mexico
ITTG S5	Chiapas, Mexico
ITTG S64	Chiapas, Mexico

* Identity according to the sequence analysis of the chromosomal gene *rpo*B.

### Nodulation tests

*Acaciella angustissima* seeds were scarified with H_2_SO_4_ for 15 min and surface sterilized with 1% (v/v) sodium hypochlorite for 10 min. Treated seeds were germinated on 0.8% agar–water plates and then placed in glass tubes filled with vermiculite moistened with Fahraeus medium (Fahraeus, 1957). Inoculation tests were also performed with *Acacia farnesiana, Leucaena leucocephala* and *Phaseolus vulgaris* cv. Negro Jamapa as described (Lloret *et al.*, 2007). Bacteria for inoculation were grown in individual PY plates and suspended in 1 mL sterile distilled water, serially diluted, and absorbance was determined at A_600 nm_. Final cell numbers were determined by plating on PY medium to count CFUs. Approximately 10^6^ bacteria mL^−1^ were added to each plant rootlet and the plants were grown in a plant growth chamber at 28 °C (Räsänen *et al.*, 2001). A negative control with uninoculated seedlings was included. After 30 days, surface-disinfected nodules were harvested and crushed in PY plates and single colonies were picked up and reinoculated for their authentication. Bacteria were conserved in 65% glycerol–PY broth and stored at −80 °C. Working cultures were maintained on YEM slants at 4 °C (Vincent, 1970).

### DNA isolation, genomic fingerprinting and DNA–DNA hybridization

Isolates were grown overnight in 2 mL PY. Total DNA was isolated and purified using the Genomic Prep™ kit (Amersham). Enterobacterial repetitive intergenic consensus (ERIC) genomic fingerprinting was obtained by PCR using primers ERIC1R and ERIC2 as described by Versalovic *et al.* (1994). The fingerprints were visually analyzed after resolution of PCR products using electrophoresis in 1.5% agarose gels loaded with half the volume of the 25 μL PCR reaction. ERIC fingerprinting was used only to confirm that the isolates analyzed were not clones or siblings (Ormeño-Orrillo *et al.*, 2006; Lloret *et al.*, 2007;). Strains showing different patterns were considered for sequencing and phylogenetic analysis. The DNA relatedness was determined using DNA–DNA hybridization experiments using ^32^P-labelled DNA of the newly proposed species (described below) *Sinorhizobium chiapanecum* ITTG S70^T^ as a probe. A filter hybridization method described previously was used (Martínez-Romero *et al.*, 1991). The amounts of DNA were standardized using integrating gel fluorescence with the Eagle Eye II system (Stratagene). anova and *t*-tests were performed to compare the percentage of DNA–DNA hybridization values among species and within species using angular transformation of percentage data (Knudsen & Curtis, 1947; Martínez-Romero & Rosenblueth, 1990;).

### PCR amplification and gene sequencing

An internal fragment of the chromosomal genes *gyrA, nolR, recA, rpoB* and 16S rRNA gene (*rrs*), and the symbiotic genes *nifH* and *nodA* were amplified using standard PCRs. Primers and annealing temperatures used for *gyrA, nolR, recA, rrs, rpoB* and *nifH* genes were performed as described in Lloret *et al.* (2007) and by Haukka *et al.* (1998) for *nodA*. Before sequencing, the amplification mixture was purified using the PCR product purification system of Roche™. The sequences generated were deposited in the GenBank public database and their accession numbers were included in the phylogenetic trees.

### Phylogenetic analysis

The protein-coding sequences were aligned using the program clustal w (Thompson *et al.*, 1994) and then aligned based on codons using dambe v4.2.13 (Xia & Xie, 2001). The alignments were edited with bioedit v5 (Hall, 1999). The best-fit evolutive models for each set of sequences were selected by the AKAIKE information criterion implemented in the modeltest v3.06 (Posada & Buckley, 2004). *rpoB* gene sequences were analyzed using the TrN+I+Γ model of evolution based on an alignment of 642 nucleotides from positions 3262 to 3903; *nodA* using the GTR+I+Γ model with 522 nucleotides from positions 67 to 588; *nifH* with the TrN+I+Γ model of evolution based on 474 nucleotides from positions 313 to 787; and for the *gyrA, recA* and *nolR* genes the model of evolution and alignment positions were as reported by Lloret *et al.* (2007). These positions were based on the *rpoB, nolR, nodA* and *nifH* genes of *Sinorhizobium meliloti* 1021 and *recA* and *gyrA* of *Agrobacterium tumefaciens* C58. The phylogenetic trees were inferred with the maximum-likelihood (ML) method using the program phyml v2.4.4 (Guindon & Gascuel, 2003) considering the α-parameter for the Gamma distribution and the proportion of invariable sites estimated by the program. For the inference of the *rrs* phylogenetic tree, *Sinorhizobium* type strains were analyzed by the neighbor-joining method (NJ) (Saitou & Nei, 1987) implemented in mega v3.1 (Kumar *et al.*, 2004) using the TrN+G model with the α-parameter for the Gamma distribution estimated with modeltest. The *rrs* phylogenetic tree was constructed using an alignment of 1417 nucleotides from positions 28 to 1444 with respect to the *rrs* gene of *S. meliloti* 1021. The topology robustness was estimated by a nonparametric bootstrap test using 100 pseudoreplicates for ML and 1000 for NJ.

### Competition assays

The nodulation capacity was evaluated in competition assays of *S. mexicanum* ITTG R7^T^ or *S. chiapanecum* ITTG S70^T^ against one randomly selected strain from each of the bacterial groups identified previously by *rpoB* gene sequence analysis. Twenty-one treatments resulted from the 12 combination mixtures plus each of the eight single strains as positive nodulation controls, and the negative control (uninoculated plants). Four replicates of inoculated plants were used per treatment. The plant growth conditions were as mentioned above for nodulation tests. The competitiveness was evaluated by the number of nodules obtained from each member of the mixture with respect to the total number of nodules. The identity of the reisolated strains was determined by plasmid patterns using the Eckhardt procedure (Eckhardt, 1978). The variation in nodule number was analyzed statistically by anova using sas software (SAS Institute Inc., 1989), followed by comparison of means by Tukey's test (*P*<0.05).

### Plant inoculation assays

The strains with the best nodulation capacity and high competitiveness were used as inoculants. Germinated seedlings of *A. angustissima* were planted in vermiculite tubes with Fahraeus medium (Fahraeus, 1957) and inoculated as described above. Plants without inoculum, with or without 30 mg KNO_3_-N per plant, served as control (Hungria *et al.*, 2001). Six replicate tubes were used per treatment and these were arranged in a completely randomized design. The plants were grown in a climate chamber at 28 °C for 90 days. At harvest, the shoot height, shoot dry weight, root dry weight and nodule number were determined, and total shoot nitrogen was assayed using the Kjeldahl method (Bremner & Mulvaney, 1982). The effect of the inoculation was analyzed statistically by anova, followed by comparison of means using Tukey's test (*P*<0.05).

## Results

### Strain identity, diversity and phylogeny

**Fig. 1:**
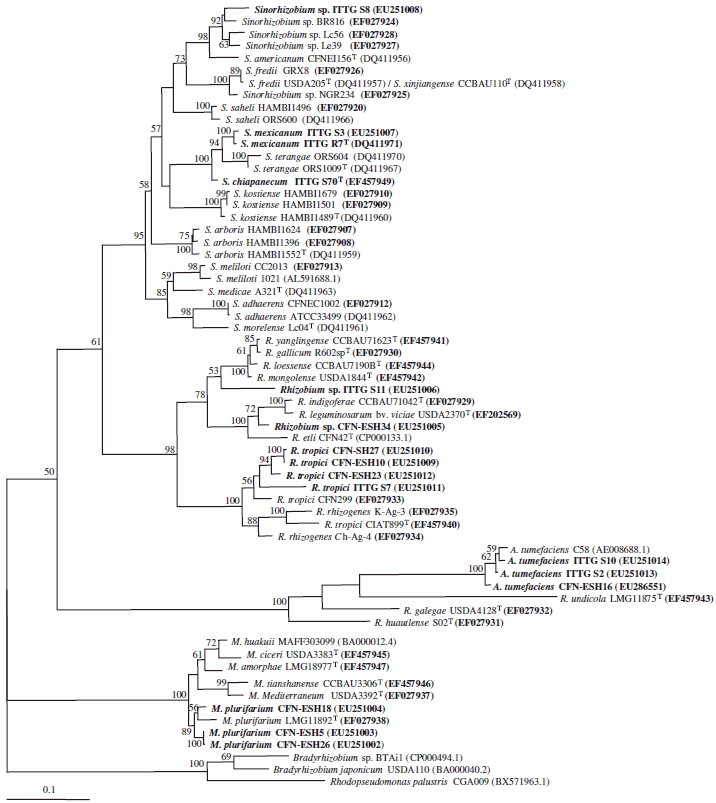

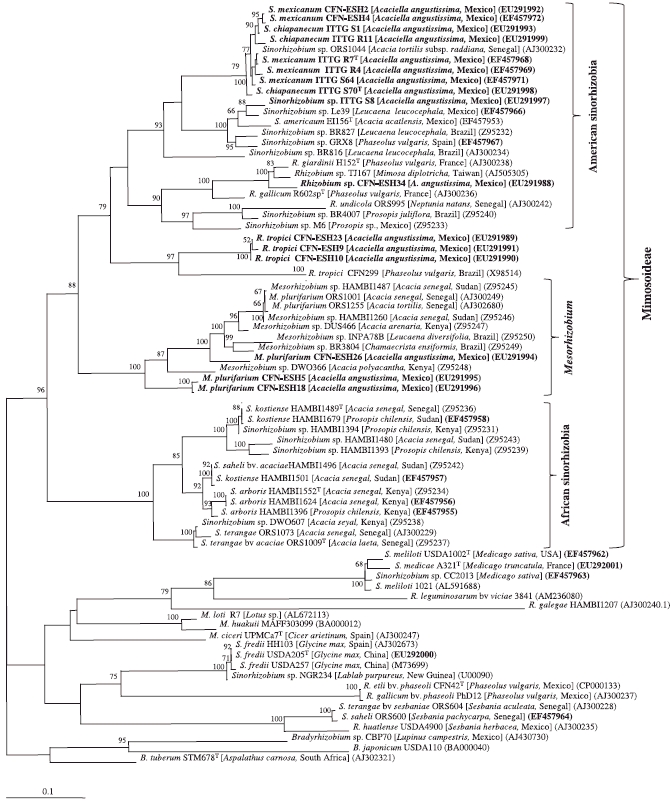
Phylogenetic tree estimated using the ML method with partial sequences of the chromosomal protein encoding gene *rpoB* using the phyml program. The alignment length was 642 nucleotides from positions 3262 to 3903 of the *rpoB* gene of *Sinorhizobium meliloti* 1021. Only bootstrap values ≥50% are shown. Type strains are indicated by superscript T. The *Acaciella angustissima* strains are shown in bold. Only haplotypes were included in each terminal branch. The accession numbers for the sequences are indicated within parentheses. Those generated in this work are shown in bold.

A total of 94 strains were obtained from *A. angustissima* root nodules in Chiapas and Morelos that were confirmed to form nodules in the original host. Thirty-eight strains that represented the different ERIC-PCR electrophoretic patterns were used for PCR amplification and sequencing. The taxonomic position of the selected strains from *A. angustissima* was determined according to the phylogenetic analysis performed with partial sequences of the chromosomal gene *rpoB*, which encodes the β-subunit of RNA polymerase (Fig. 1).

The largest percentage of isolates found at both sites corresponded to *S. mexicanum* (26.3%) while the lowest corresponded to bacteria related to *S. americanum* and *Rhizobium gallicum*, both with 2.6%. A new lineage related to *S. mexicanum* and *Sinorhizobium terangae* was isolated only in Chiapas while only the strains related to *Rhizobium etli* and *Mesorhizobium plurifarium* were found in Morelos. The largest percentage of the isolates in Chiapas corresponded to *S. mexicanum* (33.3%) and in Morelos *Rhizobium tropici* (30.0%).

**Fig. 2:**
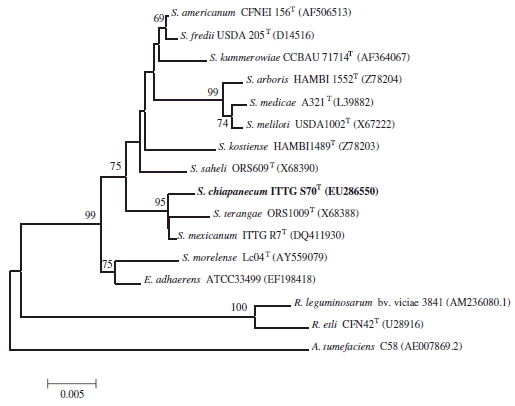
Phylogenetic tree estimated by the NJ method with the sequences of *rrs* genes using the Tamura Nei model considering gamma (α-parameter=0.9271). The alignment length was 1416 nucleotides, from positions 28 to 1444 of the *rrs* gene of *Sinorhizobium meliloti* 1021. Only bootstrap values ≥50% are shown. Type strains are indicated by superscript T. *Sinorhizobium chiapanecum* is shown in bold.

**Fig. 3:**
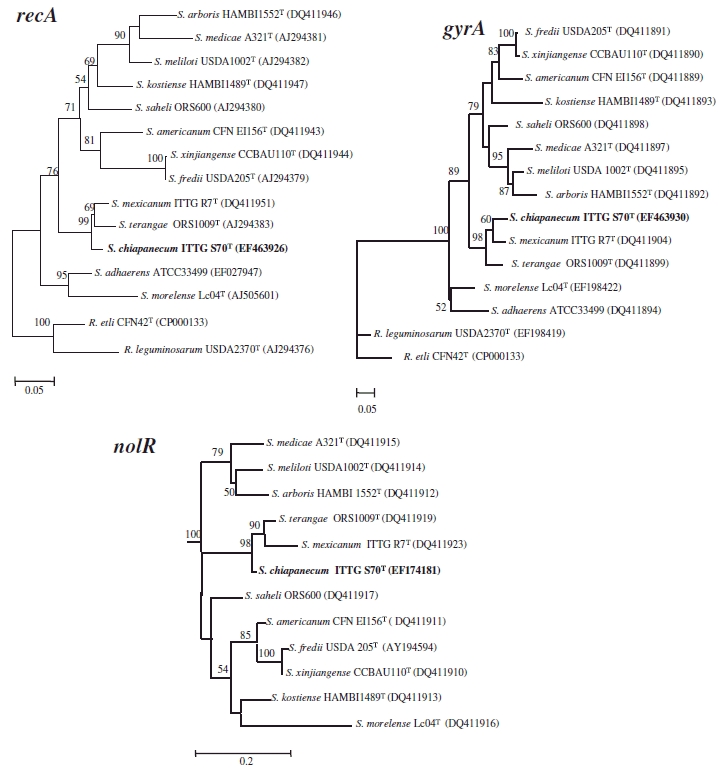
ML phylogenetic trees of partial sequences of the chromosomal protein encoding genes *gyrA, recA* and *nolR*. Type strains are indicated by superscript T. Only bootstrap values ≥50% are shown. The accession numbers for the sequences are indicated within parentheses. Those generated in this work for *Sinorhizobium chiapanecum* are shown in bold. The branches corresponding to the outgroup sequences *Rhizobium leguminosarum* USDA 2370^T^ and *Rhizobium etli* CFN42^T^ for the *nolR* tree are not shown because they were too divergent from the *Sinorhizobium* sequences.

The *Sinorhizobium* sp. strain ITTG S70^T^*rrs* gene was found to be different from all sequences available in the GenBank database and had 99% identity to its closest relative *S. mexicanum*. The phylogenetic tree with the sequences of *rrs* genes (Fig. 2) and the phylogenetic trees with *rpoB* (Fig. 1), *gyr*A, *nol*R and *rec*A genes (Fig. 3) were constructed including all of the type strains of *Sinorhizobium* species. The *recA* gene has been used previously in rhizobial phylogenetic studies (Gaunt *et al.*, 2001; Vinuesa *et al.*, 2005); *gyrA, recA, nolR* and *rpoB* were used previously to describe a new *Sinorhizobium* species (Lloret *et al.*, 2007). *gyrA* encodes the alpha-subunit of DNA gyrase, *nolR* encodes a transcriptional regulator (Chen *et al.*, 2000, 2005) and *recA* encodes the recombination protein RecA. In all phylogenetic trees, the position of strain ITTG S70^T^ as a different lineage within the *Sinorhizobium* genus was well supported. Strains ITTG R11, ITTG S68 and ITTG S71 had sequences identical to those from ITTG S70^T^ that was chosen to represent this new lineage.

Total DNA from the strain ITTG S70^T^ showed low hybridization with the strains belonging to *S. mexicanum* (<42%) and *S. terangae* (<56%), while hybridization to three strains from its own group, ITTG S68, ITTG S71 and ITTG R11 (>74%), was higher than the limit proposed for new species (70%, Stackebrandt *et al.*, 2002) (Table S2). DNA–DNA hybridization differences between *S. chiapanecum* strains and the closest species, *S. mexicanum* and *S. terangae*, were statistically significant with *P*<0.05 and *P*<0.10, respectively. It remains to be established whether similar plasmids in *S. terangae* and *S. chiapanecum* account for part of the DNA hybridization obtained. Description of species should be based on chromosomal and not plasmidic characteristics (Martínez-Romero & Jarvis, 1993). Also, phenotypic differences distinguishing *S. chiapanecum, S. terangae* and *S. mexicanum* are presented as Table S1.

The DNA–DNA hybridization, the phylogenetic position and phenotypic characteristics support that this *Sinorhizobium* lineage corresponds to a new species within the genus *Sinorhizobium*, and the proposed name is *S. chiapanecum* because it was isolated in Chiapas.

**Fig. 4:**
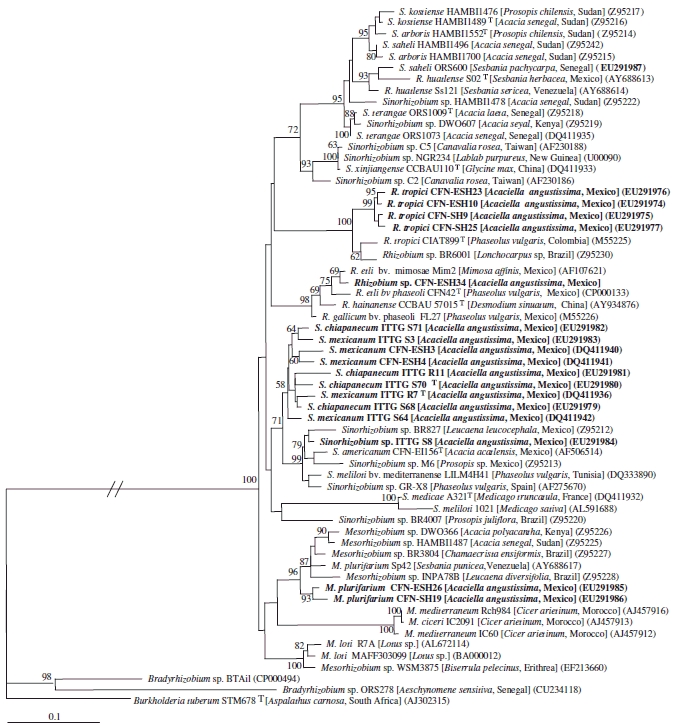
Phylogenetic tree estimated using the ML method with partial sequences of the symbiotic protein encoding *nifH* gene using the phyml program. The alignment length was 474 nucleotides from positions 313 to 786 of the *nifH* gene with respect to the *nifH* gene encoded on the pSymA of *Sinorhizobium meliloti* 1021. Only bootstrap values ≥50% are shown. Type strains are indicated by superscript T. The *Acaciella angustissima* strains are shown in bold. The accession numbers for the sequences are indicated within parentheses. Those generated in this work are shown in bold. Host and geographical origin are in parentheses.

**Fig. 5:**
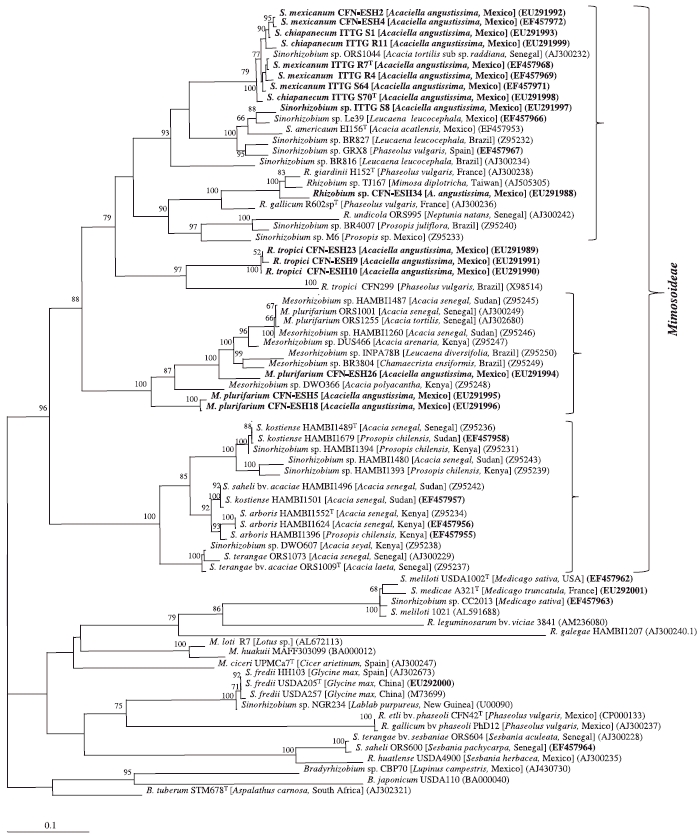
Phylogenetic tree estimated using the ML method with partial sequences of the symbiotic protein encoding the *nodA* gene using the phyml program. The alignment length was 522 nucleotides from positions 67 to 588 of the *nodA* gene with respect to the *nodA* encoded on the pSymA of *Sinorhizobium meliloti* 1021. Only bootstrap values ≥50% are shown. Type strains are indicated by superscript T. The *Acaciella angustissima* strains are shown in bold. The accession numbers for the sequences are indicated within parentheses. Those generated in this work are shown in bold. Host and geographical origin are in parentheses.

The *nifH* and *nodA* phylogenetic trees are shown in Figs 4 and 5, respectively. Symbiotic genes from the different rhizobia isolated from *A. angustissima* had affiliations with the corresponding genes from species in the genera *Rhizobium, Sinorhizobium* and *Mesorhizobium*. The phylogenies of these two symbiotic genes were incongruent with the phylogeny obtained with the chromosomal gene *rpoB*. The *nodA* sequences from *Rhizobium* sp. strains CFN ESH6 and CFN ESH34 (related to *R. etli*) isolated from *A. angustissima* were similar to the *nodA* gene of *Rhizobium giardinii* H152^T^ isolated from common bean in France, but with the *nifH* gene analysis these strains were found to be related to the *nifH* gene of *R. etli* bv. *mimosae* Mim2 isolated from *Mimosa affinis* in Mexico. The *nodA* and *nifH* genes of *R. tropici* strains CFN ESH23, CFN ESH25, CFN ESH10, CFN ESH29 and CFN ESH9 were related but not identical to *nodA* and *nifH* gene sequences from *R. tropici* CFN299 isolated from *P. vulgaris* in Mexico. The symbiotic gene sequences of *S. chiapanecum* and *S. mexicanum* isolated from *A. angustissima* clustered together and were related to a different and well-supported group that included mainly sequences from *Sinorhizobium* isolated from American legumes, among them, the strains *Sinorhizobium* sp. BR827 and BR816 from *L. leucocephala* in Brazil and *S. americanum* CFN EI156 isolated from *Acacia acatlensis* in Mexico. The *nodA* and *nifH* gene sequences from *S. terangae*, the closest relative of *S. mexicanum* according to *rpoB* gene sequences, grouped in a far distant cluster. *Mesorhizobium plurifarium* isolated from *A. angustissima* has *nodA* and *nifH* gene sequences similar to several *Mesorhizobium* species isolated from American and African hosts, mainly with the strains *Mesorhizobium* sp. DWO366 isolated from *Acacia polyacantha* in Kenya and *Mesorhizobium* sp. INPA78b isolated from *L. leucocephala* in Brazil.

### Nodulation and nodule occupancy in competition assays

Nodule occupancy evaluated from interstrain competition assays is shown in Table 2. The strains ITTG R7^T^, ITTG S70^T^, CFN ERSH34, CFN ERSH5 and ITTG S7 showed the best nodulation capacity and high competitiveness. *Sinorhizobium mexicanum* strain ITTG R7^T^ always had a greater occupancy of the nodules than the respective competing strain, ranging from 65% when combined with *Sinorhizobium* sp. ITTG S8 to 100% when combined with *Rhizobium* sp. ITTG S11. The *S. chiapanecum* strain ITTG S70^T^ did not always have a greater occupancy of the nodules than the competing strain, although this strain and *S. mexicanum* ITTG R7 were highly effective in inoculation assays with *A. angustissima* (Table 3). *Mesorhizobium plurifarium* CFN ESH5 and *Rhizobium* sp. CFN ESH34 had a greater occupancy than ITTG S70^T^ (67% and 77%), respectively. Significantly lower numbers of nodules were obtained with *M. plurifarium* CFN ESH5, *R. tropici* ITTG S7, *Sinorhizobium* sp. ITTG S8 (related to *S. americanum*), *Rhizobium* sp. ITTG S11 (related to *R. gallicum*) and *A. tumefaciens* ITTG S2, with the latter showing the lowest number of nodules and very low nitrogen fixation. All reisolated strains showed colony morphology and plasmid patterns identical to the original inoculated strains (data not shown).

**Table 3:** Effect of inoculation by the strains with high competitivity and nodulation capacity on the growth, nodulation and nitrogen fixation of *Acaciella angustissima*

Strains	Shoot height (cm)	Shoot dry weight (mg)	Root dry weight (mg)	Nodule number	Total shoot N (mg per plant)
Uninoculated	15.0 c*	76.1 b	46.3 b	0 b	30.4 c
*M. plurifarium* CFN ESH5	16.5 bc	95.0 b	40.3 a	2.1 b	39.9 c
*Rhizobium* sp. CFN ESH34	20.1 b	101.0 b	44.9 a	2.3 b	51.5 c
*R. tropici* ITTG S7	17.0 bc	96.4 b	42.9 a	2.3 b	44.3 c
*S. chiapanecum* ITTG S70^T^	24.8 a	112.9 a	44.0 a	5.3 a	101.6 b
*S. mexicanum* ITTG R7^T^	25.1 a	134.7 a	52.6 a	5.8 a	158.9 a
KNO_3_-N (30 mg per plant)	15.3 c	56.1 c	25.3 b	0 b	22.4 c

* Mean values of six replicates. The means followed by the same letter are not significantly different (*P*<0.05).

**Table 2:** Nodule occupancy by strains of *Sinorhizobium mexicanum* ITTG R7^T^ and *Sinorhizobium chiapanecum* ITTG S70^T^ and the coinoculated bacteria in competition assays in *Acaciella angustissima*

		Nodule occupancy (%) by	
Treatments	Nodule number (per plant) (±SD)*	First strain of the combination	Second strain of the combination
Uninoculated	0^h^		
*A. tumefaciens* ITTG S2	0.5 (±1.0)^gh^		
*M. plurifarium* CFN ESH5	8.0 (±1.6)^bcde^		
*Rhizobium* sp. ITTG S11	2.0 (±0.8)^fgh^		
*Rhizobium* sp. CFN ESH34	10.5 (±2.5)^abc^		
*R. tropici* ITTG S7	4.0 (±1.6)^defgh^		
*Sinorhizobium* sp. ITTG S8	2.25 (±2.1)^fgh^		
*S. chiapanecum* ITTG S70^T^	10.0 (±1.6)^abc^		
*S. mexicanum* ITTG R7^T^	14.0 (±3.7)^a^		
*S. chiapanecum* ITTG S70^T^+*A. tumefaciens* ITTG S2	4.5 (±1.3)^defgh^	61 (18)†	39
*S. chiapanecum* ITTG S70^T^+*M. plurifarium* CFN ESH5	4.5 (±2.6)^defgh^	33 (18)	67
*S. chiapanecum* ITTG S70^T^+*Rhizobium* sp. ITTG S11	4.25 (±1.7)^defgh^	82 (17)	18
*S. chiapanecum* ITTG S70^T^+*Rhizobium* sp. CFN ESH34	3.25 (±1.9)^efgh^	23 (13)	77
*S. chiapanecum* ITTG S70^T^+*R. tropici* ITTG S7	2.5 (±1.3)^fgh^	80 (10)	20
*S. chiapanecum* ITTG S70^T^+*Sinorhizobium* sp. ITTG S8	3.75 (±2.1)^defgh^	67 (15)	33
*S. mexicanum* ITTG R7^T^+*A. tumefaciens* ITTG S2	6.0 (±1.4)^cdef^	75 (24)	25
*S. mexicanum* ITTG R7^T^+*M. plurifarium* CFN ESH5	8.75 (±5.1)^abcd^	89 (35)	11
*S. mexicanum* ITTG R7^T^+*Rhizobium* sp. ITTG S11	5.5 (±1.9)^cdefg^	100 (22)	0
*S. mexicanum* ITTG R7^T^+*Rhizobium* sp. CFN ESH34	12.0 (±1.4)^ab^	71 (48)	29
*S. mexicanum* ITTG R7 ^T^+*R. tropici* ITTG S7	3.75 (±1.3)^defgh^	67 (15)	33
*S. mexicanum* ITTG R7^T^+*Sinorhizobium* sp. ITTG S8	6.5 (±3.4)^cdef^	65 (26)	35

* Mean values of four replicates. The means followed by the same letter are not significantly different (*P*<0.05).

† In parentheses, total number of nodules analyzed.

### Plant growth, nodulation and nitrogen fixation of *A. angustissima* inoculated with selected strains

The inoculation using the selected rhizobia strains had a significant effect on the growth of *A. angustissima* (Table 3). *Rhizobium* sp. CFN ESH34, *S. mexicanum* ITTG R7^T^ and *S. chiapanecum* ITTG S70^T^ had a positive effect on shoot height, shoot dry weight and root dry weight compared with the uninoculated control plants and those with added KNO_3_. Plants inoculated with these strains were on average 8.3 cm taller and weighted 109 mg more than noninoculated plants 90 days postinoculation. The number of nodules obtained with *S. mexicanum* ITTG R7^T^ and *S. chiapanecum* ITTG S70^T^ was significantly different (*P*<0.05) compared with the rest of the treatments. None of the noninoculated plants formed nodules. The plants inoculated with ITTG R7^T^ showed a significantly higher total shoot nitrogen compared with other treatments (*P*<0.05). ITTG R7^T^ was found to be the most effective strain in terms of plant growth promotion as indicated by total plant nitrogen content.

### Characteristics of *S. chiapanecum* sp. nov

*Sinorhizobium chiapanecum* (chia.pa.ne'cum. N.L. neut. adj. *chiapanecum* of Chiapas, the name of a state in Mexico where the bacterium was isolated). Gram-negative, aerobic, motile and nonspore-forming rods. Strains are fast growing and acid producers in YEM medium. The generation time for ITTG S70^T^ in YEM broth is 2.33 h at 28 °C. Colonies on PY or YEM are circular, pearly, slightly translucent and produce copious amounts of polysaccharides. Colonies are normally more than 2–4 mm in diameter within 2 days of incubation at 28 °C. The strains are resistant to nalidixic acid (120 μg mL^−1^) but not to carbenicillin (20 μg mL^−1^), ampicillin (10 μg mL^−1^) or chloramphenicol (10 μg mL^−1^). They grow in media containing 0.5%, 1.0% and 2.0% NaCl but not with 3.0% NaCl. Total DNA from strain ITTG S70^T^ showed low hybridization values with the strains belonging to *S. terangae* ORS1009^T^ (<48%) and with *S. mexicanum* ITTGR7^T^ (<33%). This species can be differentiated from other described *Sinorhizobium* species on the basis of the phylogenetic analysis of the chromosomal genes *rrs, gyrA, recA, rpoB* and *nolR*. The type strain ITTG S70^T^ was isolated from nodules of *A. angustissima* collected in the Sumidero Canyon National Park, Chiapas, Mexico. *Sinorhizobium chiapanecum* ITTG S70^T^ nodulated and fixed nitrogen in *A. angustissima, Acaciella cochliancantha, Acaciella farnesiana, Acaciella pennatula, Dolichos lablab, P. vulgaris, L. leucocephala* and *Lysiloma acapulcensis* and tolerated salinity and acidity (data not shown). ITTG S70^T^ has characteristics of the species.

## Discussion

Tropical forests in Mexico harbor many endemic plants and a high richness of species (Rzedowsky, 1978). Forests have abiotic and biotic characteristics that allow such diversity to exist. Plant speciation in Mexico seems to be driven by geographical isolation due to the complex topography of the country. The tropics have a large diversity of rhizobia (Wang *et al.*, 1999; Mohamed *et al.*, 2000; Räsänen *et al.*, 2001; Toledo *et al.*, 2003; Wolde-Meskel *et al.*, 2004;). Sinorhizobia seem to have radiated in Mexico in relation to the geographical isolation and diversity of climates, conditions and plants (Toledo *et al.*, 2003; Lloret *et al.*, 2007;). The sinorhizobia-nodulating legumes in Africa and in the Americas are considered to have had a long period of diverging evolution (Haukka *et al.*, 1998; Toledo *et al.*, 2003; Lloret *et al.*, 2007;). Our results showed that *A. angustissima* was preferentially nodulated by closely related members of the *Alphaproteobacteria*, especially sinorhizobia. Differences in symbiotic efficiency and competitiveness were found among the isolates, with *S. mexicanum* and *S. chiapanecum* strains being highly effective symbionts and good competitors. In contrast to acacias, no bradyrhizobia or *Betaproteobacteria* strains were found nodulating this legume. *Acaciella angustissima* was among the legume hosts of Latin American origin that formed nitrogen-fixing nodules with the African sinorhizobial strains *Sinorhizobium arboris* HAMBI 1552^T^, *Sinorhizobium kostiense* HAMBI 1489^T^ and *S. terangae* bv. *acaciae* ORS 1058 (Räsänen *et al.*, 2001). Tropical legumes seem to have a mild specificity when associating with nodulating bacteria (Moreira *et al.*, 1998), although under natural conditions predominant rhizobial species may be preferentially encountered in promiscuous plants (Bala & Giller, 2001; Bala *et al.*, 2003; Martínez-Romero, 2003;) as shown here.

*Mesorhizobium plurifarium* strains originally isolated from *Acacia senegal* (de Lajudie *et al.*, 1998) encompass a set of diverging strains. In this study, *M. plurifarium* strains were found in *A. angustissima* only in Morelos. In Mexico, *M. plurifarium* were found nodulating *Sesbania* (*Papilioniodeae*) trees (Wang *et al.*, 1999) and *L. leucocephala* plants grown in Morelos soils. *Acaciella* and *Leucaena* belong to the Mimosoideae subfamily of the *Leguminosae*. Plant traps with soils from Morelos were used to collect the bacteria and it has been shown that by doing so a larger diversity of bacteria may be obtained nodulating a single legume (Hungria *et al.*, 2001), and so we predicted that *Mesorhizobium* strains were the less adapted to nodulate *A. angustissima*. This turned out to be true.

We found seven isolates of *Rhizobium* similar to *R. tropici* type A, with *nod* genes more closely related (but not identical) to *nodA* of *R. tropici* than to other *nodA* genes. *Rhizobium tropici* strains are common in tropical soils and nodulate some trees from the Mimosoideae subfamily of the *Leguminosae* such as *L. leucocephala* (Martínez-Romero *et al.*, 1991) as well as *A. angustissima* (not shown).

*Rhizobium etli* is commonly isolated from *P. vulgaris* (Segovia *et al.*, 1993), but has also been isolated from other shrub legumes in Kenya (Odee *et al.*, 2002). Biovars that refer to host specificity have been described in *R. etli*. Nodulation of *Mimosoidea* plants such as *M. affinis* and *Leucaena* spp. is the characteristic of biovar mimosae (Wang *et al.*, 1999). It is probable that the *Rhizobium* sp. strains (related to *R. etli* and *R. leguminosarum*) from *A. angustissima* correspond to biovar mimosae. Strain ITTG S11 was found to be related to *R. gallicum* (Amarger *et al.*, 1997). *Rhizobium gallicum* bv. gallicum was isolated from common bean and can also nodulate *L. leucocephala* (Amarger *et al.*, 1997; Silva *et al.*, 2005;) and other species from the Mimosoideae subfamily of the *Leguminosae* (Zurdo-Piñeiro *et al.*, 2004) but it was not known that it nodulated *Acaciella*.

In addition, species of *Agrobacterium* were also found in this study. Bala & Giller (2001) reported that the legumes *Acacia auriculiformis, L. leucocephala, Gliricidia sepium, P. vulgaris* and *Sesbania sesban* formed effective nodules with one or more isolates that resembled *A. tumefaciens. Agrobacterium* strains have been isolated previously from nodules of *Acacia mellifera, A. polyacantha, Acacia nilotica* and *S. sesban* (Khbaya *et al.*, 1998; de Lajudie *et al.*, 1999;) and shrubs growing in the semi-arid and arid climates of north-western China (Tan *et al.*, 1999). Odee *et al.* (2002) indicated that agrobacteria were often found in association with root nodules as a co-occupant with rhizobia. The *Agrobacterium* strains described here were capable of forming nodules on *A. angustissima*, but the nitrogen fixation was very low. *Agrobacterium* sp. ITTG S2 (similar to *A. tumefaciens*) showed a low level of competitiveness when inoculated in competition assays. Recently, some *Agrobacterium* strains were found to be capable of forming tumors on plants as well as nodulating (Rivas *et al.*, 2004). In additional experiments, we evaluated the pathogenicity of the strains ITTG S2, ITTG S6 and ITTG S10 (all similar to *A. tumefaciens*) on sunflower plants (*Helianthus annus*) and found that these strains are not tumorogenic (not shown).

We showed that the phylogenies of the symbiotic genes were incongruent with the chromosomal genes as has been reported previously (Haukka *et al.*, 1998; Wernegreen & Riley, 1999; Laguerre *et al.*, 2001; Toledo *et al.*, 2003; Lloret *et al.*, 2007;). Symbiotic genes on elements such as plasmids and symbiotic islands are prone to lateral gene transfer (Sullivan & Ronson, 1998; Ochman & Moran, 2001;) and may be selected by hosts (Ueda *et al.*, 1995; Haukka *et al.*, 1998; Wernegreen & Riley, 1999;), as observed here, because the two species *S. mexicanum* and *S. chiapanecum* nodulating *Acaciella* have the same *nodA* genes. The three main groups described based on *nod* gene sequences (Haukka *et al.*, 1998) corresponding to African and Latin–American sinorhizobia and some *Mesorhizobium* spp. were observed in the trees presented here with several more sequences included (Fig. 5). A large group was distinguished that corresponds to *nod* genes of symbionts with the capacity to nodulate many plants from the Mimosoideae subfamily of the *Leguminosae* (Fig. 5); it is worth noticing that within this group, *R. giardinii* and *R. gallicum nod* gene sequences were included.

*Sinorhizobium* sp. ITTG S8, a strain related to *S. americanum*, clustered in the *rpoB* tree with some American strains isolated from *L. leucephala* in Mexico (Wang *et al.*, 1999) and with strain BR816 (van Rhijn *et al.*, 1994) from Brazil. This group constitutes a sister clade to *S. americanum* and could have been identified as belonging to the same species, but unpublished DNA–DNA hybridization results from our lab showed that BR816 was not a member of *S. americanum*. A new biovar has been proposed (mediterranense) (Mnasri *et al.*, 2007) to account for sinorhizobia closely related to *Sinorhizobium fredii* and with specificity for *L. leucocephala* and *P. vulgaris*. This biovar includes strain BR816 and some other strains that, despite being closely related to *S. fredii*, do not form nodules on soybean. In spite of the close relatedness of the symbiotic genes of bv. mediterranense and *S. americanum* to those from *S. mexicanum* and *S. chiapanecum*, we consider that *A. angustissima* symbionts would not correspond to biovar mediterranense because the isolates that we found to be closely related to biovar mediterranense were not efficient to nodulate *A. angustissima*, comprised only 2.6% of the original isolates and were outcompeted by *S. mexicanum* or *S. chiapanecum*.

Within the enlarged set of sequence data presented here, we observed that the *nodA* gene sequence from *Rhizobium huautlense* (not reported previously) forms a clade with other *Sinorhizobium* species nodulating *Sesbania* (Fig. 5), indicating the strong specificity for *Sesbania* nodulation and evidencing lateral transfer of symbiotic genes between *Rhizobium* and *Sinorhizobium*. The genetic coherence among symbiotic and chromosomal genes has been considered to be characteristic of rhizobia nodulating wild legumes (Wernegreen & Riley, 1999), but *S. chiapanecum* and *S. mexicanum* as well as *R. huautlense* and sinorhizobia from biovar sesbaniae, all from noncultivated hosts, do not follow this observation and show evidence of horizontal transfer of symbiotic genes.

## Statement

Reuse of this article is permitted in accordance with the Creative Commons Deed, Attribution 2.5, which does not permit commercial exploitation.
